# Meeting the health needs of displaced people fleeing Ukraine: Drawing on existing technical guidance and evidence

**DOI:** 10.1016/j.lanepe.2022.100403

**Published:** 2022-05-07

**Authors:** Bernadette N. Kumar, Rosemary James, Sally Hargreaves, Kayvan Bozorgmehr, Davide Mosca, Seyed-Moeen Hosseinalipour, Khawla Nasser AlDeen, Chrysanthi Tatsi, Reem Mussa, Apostolos Veizis, Daniela Kállayová, Karl Blanchet, Rita Sá Machado, Miriam Orcutt, Santino Severoni

**Affiliations:** a Norwegian Institute of Public Health, Norway; b Lancet Migration European Regional Hub, Switzerland; c Geneva Centre of Humanitarian Studies, Geneva, Switzerland; d University Hospitals of North Midlands NHS Trust, Stoke-on-Trent, UK; e The Migrant Health Research Group, Institute for Infection and Immunity, St George's, University of London, London, UK; f Department of Population Medicine and Health Services Research, School of Public Health, Bielefeld University, Bielefeld, Germany; g Department of General Practice and Health Services Research, Section for Health Equity Studies and Migration, University Hospital Heidelberg, Heidelberg, Germany; h Realizing Health SDGs for Migrants, Displaced and Communities, Switzerland; i SOS Children's Villages Greece, Athens, Greece; j Humanitarian Affairs Adviser, Forced Migration, Médecins Sans Frontières, Operational Centre of Brussels, Brussels, Belgium; k Ministry of Health, Slovak Republic; l INTERSOS Hellas, Thessaloniki, Greece; m Health and Migration Programme, Office of the Deputy Director-General, World Health Organization, Geneva, Switzerland

**Keywords:** Ukraine, Health Needs, Refugees, Technical Guidance

## Abstract

The invasion of Ukraine has unleashed a humanitarian crisis and the impact is devastating for millions displaced in Ukraine and for those fleeing the country. Receiving countries in Europe are reeling with shock and disbelief and trying at the same time to grapple with the reality of providing for a large, unplanned, unprecedented number of refugees mainly women and children on the move. Several calls for actions, comments and statements express outrage, the risks, and the impending consequences to life and health. There is a need to constantly assess the situation on the ground, identify priorities for health and provide guidance regarding how these needs could be addressed. Therefore, the Lancet Migration European Regional Hub conducted rapid interviews with key informants to identify these needs, and in collaboration with the World Health Organization Health and Migration Programme, summarized how these could be addressed. This viewpoint provides a summary of the situation in receiving countries and the technical guidance required that could be useful for providing assistance in the current refugee crisis.

## Background

European migratory flows, whether forced displacements (Syria, Balkans, Ukraine^1^), or voluntary, are not new. Until the war, Ukraine has been among the top ten destination countries for migrants worldwide and increasing in recent years, especially to neighbouring countries and the Balkans.^2^ As the 2022 Ukrainian crisis evolves, millions have been displaced and will continue to grow, with countries such as Poland, Romania, Moldova, Hungary, and Slovakia currently managing the highest influx of refugees arriving directly at household, communities as well as at reception centres. In Europe, though health systems’ responsiveness to migration has improved, huge differences in terms of preparedness remain, and health agencies capacity to address needs of refugees stretched. Our aim through this paper is to provide a better understanding of the rapidly evolving situation in hosting border countries, to identify immediate and short-term health needs and to highlight guidance around how to address these needs. Additionally, we conducted nine key informant interviews, with NGO field staff and health authorities in six receiving countries and within Ukraine (Table 1) to triangulate with information from the ground.

**Table 1:** Key informants interviewed (throughout March 2022).

Interview Number	Country	Organization
1	Greece	INTERSOS
2	Moldova	INTERSOS
3	Slovakia	Ministry of Health
4	Germany	Médecins du Monde Germany
5	Czech Republic	National Institute of Public Health of Czech Republic
6	Hungary	Médecins Sans Frontières
7	Ukraine	Ministry of Health
8	Ukraine	International Medical Corps
9	Poland	The Maria Grzegorzewska University

## Situation on the ground

The Temporary Protection Directive (TPD) issued by the European Union (EU) provides rights to basic protection including access to medical treatment for all Ukrainian citizens and their family members, as well as basic needs to non-European nationals^3^ While the TPD is welcome by several countries, and communities and households are positive to refugees, eligibility criteria and implementation delays may lead to exclusions of protection, access to healthcare, accommodation work and education, including people who fled Ukraine prior to February 24.^4^ Over 90% of the fleeing population are women and children including unaccompanied minors. The pre-conflict health situation in Ukraine^5^ indicates that while the disease burden might be similar to the other countries in the region, concerns such as low childhood and COVID-19 vaccination rates will require additional attention.^6^ Internally displaced populations within Ukraine, from the ongoing conflict, may have higher rates of mental distress, non-communicable diseases, and be exposed to certain infectious diseases, due in part to lack of access to medicines, healthcare and poor living conditions.^7^ People with existing vulnerabilities are at higher risk of adverse health outcomes. Summarized below, are the key evolving health risks as per the key informant interviews in receiving countries (Table 2).

**Table 2:** Key Evolving Health Risks and Challenges among Refugees Fleeing Ukraine and Urgent responses required (*technical guidance re. responses are provided in the reference list*).

Health Risks and Challenges	Urgent Responses Needed
Maternal and newborn health: Interruption of antenatal and obstetric care	Emergency maternal and new-born- care^8^
Undernutrition of women and children	Food, nutritional supplements, and cash distributions
Continuity of Care - medicines and treatments	Chronic conditions (cardiovascular, diabetes) and existing infectious diseases, specifically TB^9^/ HIV^10^ Access to a healthcare professional^11^ Assess immediate needs, Access to essential medicines and medical supplies,^12^
Cold climate and long journeys- upper respiratory infections, pneumonia, frostbite, and hypothermia	Provision of shelter, clothes, blankets Treatment of common ailments
Injuries and trauma, including mass casualties	First aid,^13^ including training of community volunteers in triage and first response, medical evacuations; Medical equipment and supplies
Vaccination	Routine childhood immunisations,^14^ catch-up vaccination in older cohorts (specifically MMR and polio); COVID-19 vaccines and boosters^15^, ^16^, ^17^
Mental distress and trauma of displaced including Syrian, Iraqi and Afghani asylum seekers (double displacement)	Psychological first aid,^18^ Culturally-sensitive accessible mental health services,^19^^,^^20^ mhGAP,^21^ Community-based MHPSS programs,^20^ Support with basic needs to regain safety and security^22^
Lack of safe water and hygiene, and confined living environments, risking disease outbreaks, including the spread of COVID-19	Supplies and equipment to maintain infection prevention^23^ and control measures; maintenance of vaccination, screening, and surveillance programmes
Sexual violence and trafficking	Protection, safeguarding, and psychosocial support Sexual and reproductive health services,^24^^,^^25^ including for adolescents,^26^ dignity kits,^27^ gender-based and intimate partner violence & rape^28^ clinical management, and safe termination of pregnancy^29^

Abbreviations: TB: Tuberculosis, HIV: Human Immunodeficiency Virus, MMR: Measles, Mumps and Rubella, mhGAP: Mental Health Gap Action Programme, MHPSS: Mental Health and Psychosocial Support.

The health workforce in receiving countries might become strained as a result of the challenge of coping with unfamiliar cultural needs, language barriers, lack of medical supplies, equipment, blood products, and essential medicines. Additional support to the health workforce by respective authorities, as well as from civil society and patient advocacy initiatives, including competencies training and strengthening of essential public health operations, is required.^30^^,^^31^

## Humanitarian and health system response to date

There is already a wealth of resources, guidelines (as indicated in Table 2), and systems in place that enhance Europe's preparedness to respond^32^ to the needs of communities displaced from Ukraine. According to colleagues on the ground, it has been helpful to have access to a series of educational material for humanitarian organizations via online platforms. In addition to the table above, guidance specific to forced displacement from Ukraine; ECDC guidance on prevention and control of infectious disease,^6^^,^^33^, ^34^, ^35^ as well as WHO guidance and situation analysis and reports^36^, ^37^, ^38^ have been produced. The national health systems, humanitarian agencies, and UN organizations are currently providing urgent health care and essential supplies. Initial responses on the ground were led by the International Committee of the Red Cross, Caritas and other local NGOs along with Ukrainian diaspora networks. In some settings, cash transfers are being used to provide food, water, and other essential supplies.^39^ The UNHCR and UNICEF in collaboration with WHO^38^ are establishing safe spaces with access to protection services known as “Blue Dots” in six countries (Belarus, Czech Republic, Hungary, Moldova, Poland, Romania and Slovakia).^40^ WHO and UNHCR are to co-chair a Mental Health and Psychosocial Support (MHPSS) working group in Poland.^41^ UHNCR is coordinating an interagency Regional Refugee Response Plan (RRP), including a Protection from Sexual Exploitation and Abuse Taskforce^42^ and Regional Cash Working Groups.^43^ Host countries are using existing mechanisms from past experiences to operationalize the EU directive to provide access to health care services.^44^^,^^45^ Additional staff are being deployed, from ministries of health as well as NGOs. Some host countries are accelerating recognition of Ukrainian medical qualifications, to support their own health workforce and provide employment to refugees.^46^^,^^47^

## Challenges on the ground

Early-stage observations include ineffective coordination, lack of health system preparedness, and inadequate cross-border collaboration. Health response teams require additional financial and human resources. Colleagues report satisfaction with existing available health guidance however implementing and operationalizing these remains a challenge. Some NGOs reported that more tools and training on triage are needed, to enable rapid prioritization of health, especially at borders and reception centres. Overcrowding, shared accommodation, and long queues, on initial points of entry limit the enforcement of public health measures against the spread of infectious disease, especially SARS-CoV-2. Some report that existing refugees are being moved from their housing/reception centres to worse conditions, in order to make room for people fleeing Ukraine.^48^ Concerns of sexual and gender-based violence are arising, given that the majority of refugees to date are women and children, many unaccompanied. In some cases, they are being welcomed directly from their point of arrival to host families/into the community, with little or no vetting and protection. Informants expressed concern about the availability of culturally sensitive MHPSS services, many countries have asked for additional MHPS. While these initiatives can offer initial support, it is insufficient for the needs of displaced populations. Safe corridors, mechanisms for referral to higher healthcare services, and integration into the health systems of host countries needs to be addressed. This is particularly important for people requiring tertiary care, for example, dialysis^49^ and chemotherapy^50^, ^51^, ^52^ patients.

## Strengthening the public health and humanitarian response

We have brought together reflections and voices that offer a better understanding of the situation on the ground including responses required to meet the health needs. Robust quantitative and qualitative analyses are now needed, to understand and predict needs of the fleeing population, and to enable an evidence-informed response, including rapid health needs assessments. It is crucial to monitor health conditions at points of entry, inside reception centres, and upon integration into the community, and relay this information to health authorities for surveillance, response, and continuity of care. Timely and coordinated referral mechanisms should be in place, ensuring continuity of care and free rapid access to healthcare and health services, to maintain immediate and medium-term assistance to patients and their families. Countries must also prepare for the integration of arriving refugees into their health systems. In doing so, it is important to avoid the impact on existing refugees, to not de-prioritize other vulnerable populations. Some medicines will be in greater demand, such as antibiotics to treat multi-drug resistant TB, and hepatitis medicines. Governments and International organizations must act quickly to source supply and issue prescribing guidelines that are inclusive. The right to universal health coverage should be upheld, and restrictive policies on access to SRHR services re-evaluated.

We must also look beyond immediate health challenges, and work holistically, to improve social determinants of health, such as access to financial aid, safe housing, employment, and schooling. Member states and the EU should act swiftly to increase emergency aid, but also to invest in sustainable health and social care systems of the countries neighbouring Ukraine that are undoubtedly going to be strained in terms of resources. Inclusion and representation of refugee and migrant healthcare workers- can fill-in service gaps. To provide livelihoods and promote integration, the rapid recognition of Ukrainian healthcare qualifications by host countries would be beneficial.^47^^,^^52^

As observed with humanitarian health guidance generated during the onset of the COVID-19 pandemic, it is essential that existing guidelines are adapted and contextualized, taking into consideration factors such as cultural and geographical differences, available and missing resources, and language needs, among others. For instance, health authorities can translate, contextualize, and adapt health communication messages, capitalizing on the support of existing diaspora/members of the health workforce who already reside in these countries.

Countries should establish safe and dignified reception centres and community hosts for all refugees including those fleeing Ukraine. We ask for strong leadership to strengthen inter-agency communication and coordination (i.e., through the RRP) as it is vital to ensuring a coordinated and effective response and knowledge-sharing.^53^ Assistance to newly arrived displaced people should by no means entail a reduction in humanitarian protection for other categories -additional resources need to be mobilized. Coordination, that hinders duplication of action and wastage of resources including cross-border collaboration, is essential during crises. Sharing unified, contextualised guidance between NGOs, Ministries of Health, and other health system stakeholders in countries and across borders will prevent reinventing the wheel and save time and resources.

Mobility trends indicate that no country can afford to ignore migration and enhancing migration governance should be a priority. The migration discourse must move beyond politicization so that systems are better prepared to manage migration. We commend all host countries in rising to the challenges and supporting a human rights-based approach, including equitable access to universal health coverage, for all people fleeing Ukraine and beyond. We reiterate a well-coordinated response using the existing tools and resources, as well as adaptation of these for the existing context. In the longer term, it will be important to keep the momentum to ensure sustainable and responsive systems to migrant and refugee health. Ongoing learning and implementation of available evidence and guidance is key to a cohesive response that puts people first with their health and wellbeing at the forefront.

## Contributors

The Lancet Migration European Regional Hub is co-hosted by KB of the Geneva Centre of Humanitarian Studies and BK, Lancet Migration co-chair. BK conceptualised the piece, drafted the outline, supervised the project, reviewed the data collected, drafted, reviewed, and edited all drafts including the final draft. RJ assisted with the methodology, data collection, drafted, reviewed, and edited all versions. RJ, SMH, KNA, and CT conducted the field interviews. KB supervised the data collection. MO, RM and SS provided inputs regarding the responses required and technical guidance. All authors reviewed and edited the piece. All authors accept responsibility for this article.

## Funding

None to declare.

## Declaration of Interests

None to declare.
